# Isolation of Cellulose Degrading Fungi from Decaying Banana Pseudostem and* Strelitzia alba*

**DOI:** 10.1155/2019/1390890

**Published:** 2019-07-25

**Authors:** L. M. Legodi, D. La Grange, E. L. Jansen van Rensburg, I. Ncube

**Affiliations:** University of Limpopo Department of Biochemistry, Microbiology and Biotechnology, Private Bag X 1106, Sovenga, 0727, South Africa

## Abstract

Cellulases are a group of hydrolytic enzymes that break down cellulose to glucose units. These enzymes are used in the food, beverage, textile, pulp, and paper and the biofuel industries. The aim of this study was to isolate fungi from natural compost and produce cellulases in submerged fermentation (SmF). Initial selection was based on the ability of the fungi to grow on agar containing Avicel followed by cellulase activity determination in the form of endoglucanase and total cellulase activity. Ten fungal isolates obtained from the screening process showed good endoglucanase activity on carboxymethyl cellulose-Congo Red agar plates. Six of the fungal isolates were selected based on high total cellulase activity and identified as belonging to the genera* Trichoderma* and* Aspergillus*. In SmF of synthetic media with an initial pH of 6.5 at 30°C* Trichoderma longibrachiatum* LMLSAUL 14-1 produced total cellulase activity of 8 FPU/mL and endoglucanase activity of 23 U/mL whilst* Trichoderma harzianum* LMLBP07 13-5 produced 6 FPU/mL and endoglucanase activity of 16 U/mL. The produced levels of both cellulases and endoglucanase by* Trichoderma* species were higher than the levels for the* Aspergillus fumigatus* strains.* Aspergillus fumigatus* LMLPS 13-4 produced higher *β*-glucosidase 38 U/mL activity than* Trichoderma* species.

## 1. Introduction

Cellulose is a simple linear organic polymer of *β*-1-4 linked glucopyranose units with varying degrees of polymerization (DPs). In primary cell walls the DP range is 5000 – 7500 glucopyranose units, whereas the DP in secondary cell walls is approximately 10000 and 15000 [1]. This polysaccharide is abundantly available in materials, such as agro-wastes, municipal wastes, and forest residues. The hydrolysis of cellulosic biomass through enzymatic reaction is a preferred method over chemical reaction due to the absence of sugar degradation in the enzymatic process [2].

Cellulases are enzymes with different specificities to catalyse the hydrolysis of glycosidic bonds within cellulose (Jecu, 2000; Khan* et al*., 2016). The enzymatic hydrolysis of cellulose involves exoglucanases (exo-1,4-*β*-glucanases, EC 3.2.1.91) endoglucanases (endo-1,4-*β*-glucanases, EC 3.2.1.4) and *β*-glucosidases (*β*-D-glucoside glucohydrolase, EC 3.2.1.21) which exhibit high specificity for the *β*-1.4 glycosidic linkages (Jecu, 2000). Total cellulase is an activity involving the synergistic actions of the above enzymes that is measured using insoluble substrates such as the Whatman No. 1 filter paper, cotton linter, microcrystalline cellulose (Avicel), or bacterial cellulose [2].

The mechanism of action reveals that endoglucanases cleaves cellulose internally at the *β*-1-4 glycosidic linkage releasing oligosaccharide chains of different lengths, whereas exoglucanases cleaves the oligosaccharide chains from either the reducing or nonreducing ends to release disaccharides tri- and tetrasaccharides. *β*-Glucosidase cleaves the *β*-1-4 linkage in the disaccharide (cellobiose) to release glucose molecules and it can also hydrolyse very short chain *β*-1,4 oligoglucosides up to cellohexaose, but the reaction rate decreases with chain length ([3, 4]; Saini* et al*., 2015).

Cellulases are increasingly demanded for use in the biofuel industry to produce bioethanol from cellulosic biomass. Technology for the utilization of cellulosic biomass for the production of bioethanol is progressing slowly due to high cost of cellulases production, the recalcitrant nature of cellulosic biomass, and inefficiency of available cellulases to release more fermentable sugars [5]. Research efforts have been undertaken to improve the catalytic efficiency of the known enzymes, identify new enzymes, and optimise enzyme mix preparations for cellulosic biomass hydrolysis [6]. Improving fungal hydrolytic activity and finding stable enzymes that are tolerant to extreme conditions have become priority [7].

Agricultural waste such as banana by-products provides an alternative to the use of food crops such as corn and wheat in ethanol production [8]. During the harvest of banana, waste generated including leaves, rachis, and pseudostem, all contain high levels of cellulose [9, 10]. These cellulose rich materials are discarded and left to decay either in the plantation site or at a dumping site [10]. The decaying of plant material is facilitated by cellulolytic microorganisms. Hence this study aimed to isolate fungi that secrete cellulases with ability to hydrolyse cellulose in banana pseudostem to fermentable sugars for the bioethanol industry.

## 2. Materials and Methods

### 2.1. Sample Collection and Screening

Upper soil samples were collected in clean plastic bags from decomposed banana agro-waste (dumpsite and plantations) at the Tzaneen Allesbeeste farm, Limpopo, South Africa and from decomposed* Strelitzia alba *plant at the University of Limpopo campus, South Africa. Fungal isolation was done by serial dilution method. Three-gram samples of soil from each site were suspended in 50 ml sterile distilled water. A 100 *μ*l aliquot of soil suspension that had been diluted ten times was spread plated onto a selective Avicel agar medium (2% Avicel, 1.5% agar, 5 ml chloramphenicol (50 mg/ml) and 0.67% Yeast Nitrogen Base (YNB) without amino acids. The Avicel agar plates were incubated at 30°C until fungal growth was evident. The fungi were purified by hyphal tip method [11, 12]. The fungal isolates were maintained on malt extract agar (30 g malt extract, 20 g dextrose, 3 g peptone, and 15 g agar) at 4°C.

The enzymatic screening for cellulases was based on a carboxymethyl cellulose (CMC) plate assay whereby the agar medium was mixed with Congo Red (CR) dye [13]. The composition of the medium was 1% CMC, 1.5% agar, 0.67% YNB without amino acids, and 0.01% Congo Red. Ten fungal isolates obtained were cultured on CMC-CR agar and incubated at 30°C for 72-96 h. As growth develops on CMC-CR agar plate, the hydrolysis of CMC releases the bound CR dye. This is revealed by the appearance of pale yellow halo zone surrounding the fungal colony.

The second screening was based on cellulolytic hydrolysis of filter paper (total cellulase activity). Six fungal isolates were cultured in a synthetic medium described by Peixoto [14] and Avicel as the sole source of carbon. The synthetic media consisted of 2 g/l K_2_HPO_4_, 0.5 g/l KCl, 0.01 g/l FeSO_4_.7H_2_O, 20 g/l Avicel, 0.15 g/l MgSO_4_.7H_2_O, 7 g/l KH_2_PO_4_, 1 g/l (NH_4_)SO_4_, and 1 g/l yeast extract. The pH of the medium was adjusted to 5.5 prior to sterilization. Inoculation was done by cutting approximately 0.5 × 0.5 cm of fungal growth on agar medium and adding it into 100 ml medium in 250 ml Erlenmeyer flasks. All the flasks (Triplicates) were incubated at 30°C for 7 days, while shaking at 150 rpm. A 5 ml sample was removed after every 24 h of incubation and used for determining total cellulase activity as expressed in filter paper units (FPU).

### 2.2. Molecular Identification of Fungal Isolates

Identification of fungi was based on the PCR amplification of the conserved nucleotide sequence of ribosomal internal transcribed spacer (ITS) of the gene coding for the 18S, 5.8S, and 28S rRNA [15]. Isolation and purification of fungal genomic DNA was performed by following the procedure outlined in the ZR Fungal/Bacterial DNA Kit™ (Zymo Research, Catalogue No. D6005).

The ITS target region (i.e., ITS-5.8S-ITS fragment) was amplified using EconoTaq® PLUS GREEN 2X Master Mix (Lucigen,) using the following primers, ITS1 5′-TCCGTAGGTGAACCTGAGG-3′ and ITS4 5′-TCCTCCGCTTATTGATATGC-3′ [15, 16]. The following PCR conditions were used: 35 cycles including an initial denaturation step at 95°C for 2 minutes. Subsequent denaturation was at 95°C, 30 seconds, annealing at 50°C for 30 seconds, and extension at 72°C for 1 minute. A final extension at 72°C for 10 minutes was followed by holding at 4°C. The PCR products were analysed on a 1% agarose gel.

DNA sequencing was done using ABI V3.1 Big dye according to manufacturer's instructions on the ABI 3500 XL Genetic Analyzer (Applied Biosystems, ThermoFisher Scientific Species were identified by searching databases using BLAST (http://www.ncbi.nlm.nih.gov/BLAST/).

### 2.3. Effect of Initial Medium pH and Incubation Temperature on Cellulase Production

The effect of initial pH of the media on the production of cellulases was studied by adjusting the pH of the media using either 1 M NaOH or 1 M HCl solutions to pH values in the range of 4.5 to 7.0. The effect of temperature on the production of cellulase was investigated at 30, 35, and 40°C with pH being adjusted to 6.5 or 7.0 based on cellulase activity determined during pH studies. Inoculation and incubation conditions were maintained as described above, in secondary screening section. Samples of 5 m l were harvested every 24 h of incubation and used for enzyme assays. Crude enzyme was prepared by centrifugation of harvested sample using a Beckman Coulter® microfuge® 16 centrifuge at 13000 rpm for 10 min at room temperature.

### 2.4. Cellulase Activity Assay

The total cellulase activity was determined by filter paper assay (FPase) using Whatman No. 1 filter paper strip with a dimension of 1 × 6.0 cm equivalent to 50 mg of substrate according to Ghose (1987). At least two dilutions were made, one dilution that release slightly less than 2.0 mg and the other dilution releasing more than 2.0 mg. The reaction mixture contained 1.0 ml of 0.05 M Na-citrate, pH 5.0, filter paper strip, and 0.5 ml of crude enzyme diluted accordingly. The mixture was incubated at 50°C for 1 h. The released reducing sugar was estimated by addition of 3,5-dinitrosalicylic acid (DNS) with glucose as standard. The absorbance was read at 540 nm by using Beckman Coulter, DU® 720 UV/Vis spectrophotometer. The assay was performed in triplicate including controls. Filter paper activity (FPU) is defined as 0.37 divided by the amount of enzyme required to liberate 2.0 mg of glucose from filter paper strip (≈ 50 mg) in 1 h.

### 2.5. Endoglucanase Assay

Endoglucanase activity in the culture supernatant was determined according to the method described by Ghose (1987). The reaction mixture contained 0.5 ml of 1% CMC in 0.05 M Na-acetate buffer, pH 5.0, and 0.5 ml of appropriately diluted crude enzyme. The mixture was incubated at 50°C for 30 min and the released reducing sugar was estimated as indicated in the assay for total activity above. One unit of endoglucanase activity was defined as the amount of enzyme liberating one *μ*mole of reducing sugar from CMC under the assay conditions.

### 2.6. *β*-Glucosidase Assay

*β*-Glucosidase activity was determined according to the method described by Herr [17]. The reaction mixture contained 0.2 ml of 0.01 M* ρ*-nitrophenyl *β*-d-glucopyranoside (pNPG) in 0.05 M citrate buffer pH 4.8 and 0.2 ml of appropriately diluted enzyme solution. The substrate control contained 0.4 ml of 0.01 M pNPG prepared in 0.05 M citrate buffer at pH 4.8. The mixtures were incubated at 50°C for 30 min. The reaction was stopped by adding 4 ml of a 50 mM NaOH-Glycine buffer, pH 10.6. The activity of the enzyme, indicated by the release of *ρ*-nitrophenol, was determined at 420 nm using Beckman Coulter, DU® 720 UV/Vis spectrophotometer [18, 19]. One unit of *β*-glucosidase activity was defined as the amount of enzyme liberating one *μ*mole of* ρ*-nitrophenol under the assay conditions.

### 2.7. Calculations of Enzyme Activity

Cellulase activity was determined by filter paper assay using Whatman No. 1 according to Ghose (1987).

#### 2.7.1. Cellulases (FPase)

(1) $$\begin{array}{c} FPA\left(\;\frac{FPU}{mL}\;\right)=\frac{0.37}{\left[Enz\right]}, \end{array}$$

where [Enz] is the concentration of enzyme that releases 2.0 mg of glucose from filter paper in 60 minutes.

#### 2.7.2. Endoglucanase (CMCase) and *β*-Glucosidase

To estimate the activities of either endoglucanase and/or *β*-glucosidase based on the released reducing sugars or* ρ*-nitrophenol, the following equation was used [20]:

(2) $$\begin{array}{c} \beta -glucosidase\;\;or\;\;CMCase\;\;\left(\;\frac{U}{mL}\;\right)=\frac{∆E\times Vf\times Df}{\varepsilon \times ∆t\times Venz}, \end{array}$$

whereby ΔE is absorbance value at 540 nm, Vf is final volume, *ε* is extinction coefficient of glucose (slope), Δt is incubation time, Venz is volume of crude enzyme, and DF is dilution factor (if applicable).

## 3. Results

Ten fungal isolates were obtained based on their ability to grow on Avicel as sole source of carbon on solid media. These fungal isolates were further screened for endoglucanase activity by culturing on CMC-CR agar plates at 30°C for 72 - 96 h. The results revealed that all the isolated fungi were able to grow and secrete endoglucanase which hydrolysed CMC bound to CR dye. This was revealed by appearance of pale-yellow “halo zone” around the fungal growth or colony and it was indication of endoglucanase activity or CMC hydrolysis. However, only six fungal isolates indicated larger halo zones around growth. These six fungal isolates were subsequently identified using ITS sequencing. The identification of the selected six isolates revealed two different genera, namely,* Trichoderma* and* Aspergillus *(Table 1). The* Trichoderma* species were* T. longibrachiatum* and* T. harzianum* and the* Aspergillus* species were* A. fumigatus*. The six fungal isolates were further evaluated for cellulase production in submerged fermentation.

**Figure 1:**
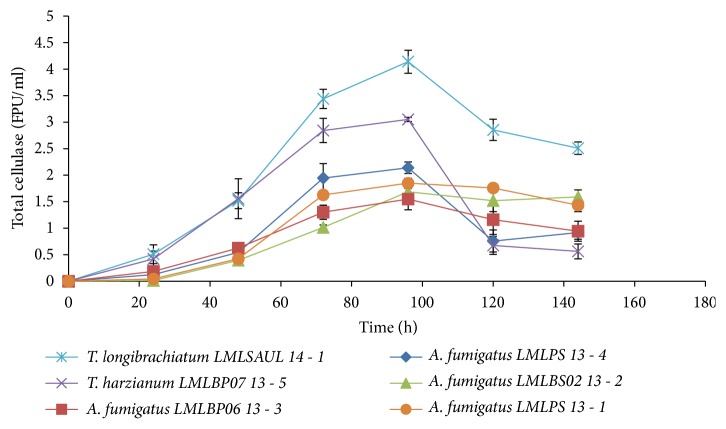
Time course for cellulase production by isolated fungal strains at 30°C and initial pH 5.5.

A quantitative evaluation of cellulase production by the selected fungi (Table 1) was carried out in submerged fermentation using Avicel as a substrate at an initial pH of 5.5 at 30°C. The production levels of cellulases were measured by the activity of the enzymes under specified conditions. Maximum cellulase activity was observed after 96 h for all the fungal strains (Figure 1).* T. longibrachiatum* LMLSAUL 14-1 produced 4.1 FPU/ml followed by* T. harzianum* LMLBP07 13-5 with activity of 3.1 FPU/ml, A.* fumigatus* LMLPS 13-4 with activity of 2.1 FPU/ml,* A*.* fumigatus* LMLPS 13-1 with activity of 1.9 FPU/ml, A.* fumigatus* LMLBS02 13-2 activity of 1.7 FPU/ml, and* A. fumigatus* LMLBP06 13-3 activity of 1.6 FPU/ml.

**Figure 2:**
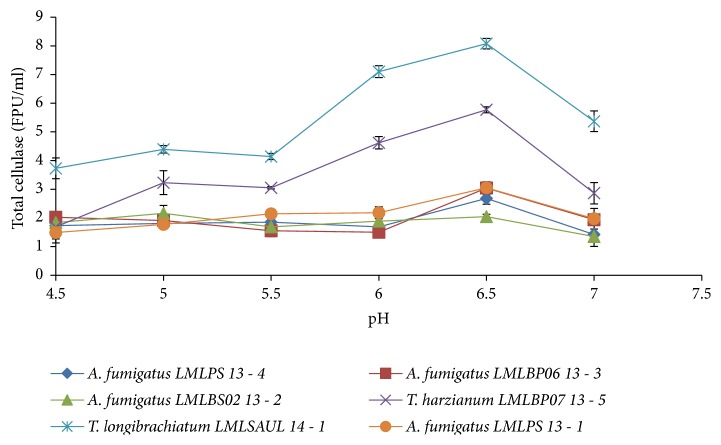
The effect of initial medium pH on the production of cellulases by isolated fungi at 30°C.

The influence of pH on the production of cellulase was assessed for the six fungal strains at 30°C (Figure 2). Maximum cellulase activity was detected for all fungal isolates when the initial pH of the medium was 6.5 (Figure 2). The* Trichoderma longibrachiatum *LMLSAUL 14-1 strain produced the highest total cellulase activity of 8.1 FPU/ml. This was followed by* T*.* harzianum* LMLBP07 13-5 with activity of 5.8 FPU/ml.* A. fumigatus *LMLBP06 13-3 and* A. fumigatus* LMLPS 13-1 produced maximum cellulase activity of 3.1 FPU/ml with the lowest cellulase activity of 2.0 FPU/ml being produced by* A. fumigatus *LMLPS 13-4.

**Figure 3:**
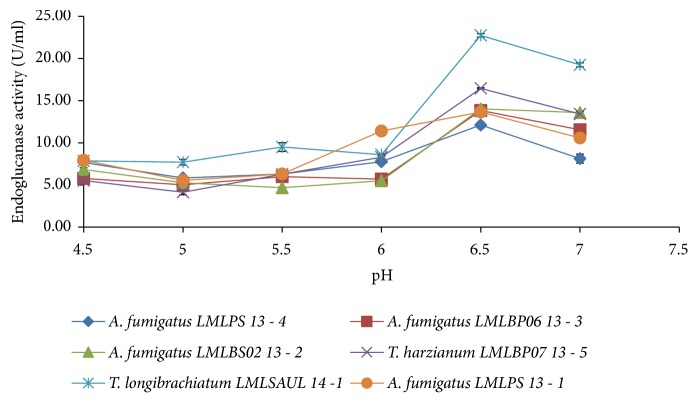
The effect of initial medium pH on the production of endoglucanases by the isolated fungal strains at 30°C.

The production of endoglucanase activity was highest at pH 6.5 for all the fungal strains (Figure 3).* T. longibrachiatum *LMLSAUL 14 - 1 and* T. harzianum *LMLBP07 13-5 produced activities of 23.0 U/ml and 16.0 U/ml, respectively, whereas* A. fumigatus* LMLPS 13-1, LMLBS02 13-2, and LMLBP06 13-3 produced amounts of 14.0 U/ml. The lowest activity of 12.1 U/ml was noted for* A. fumigatus* LMLPS 13-4.

**Figure 4:**
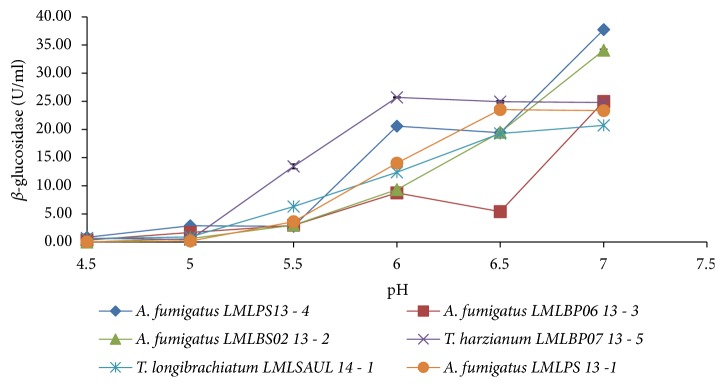
The effect of initial medium pH on the production of *β*-glucosidase by the isolated fungi at 30°C.

The levels of *β*-glucosidase produced with respect to initial medium pH differed between the fungal strains.* A. fumigatus* LMLPS 13-4 and* A. fumigatus* LMLBS02 13-2 produced higher activities of 38.0 U/ml and 34.1 U/ml at pH 7.0, respectively, compared to the other isolates tested (Figure 4). Some fungi were able to secrete higher *β*-glucosidase at various pH values.* T. harzianum* LMLBP07 13-5 produced *β*-glucosidase activity of 25.7 U/ml over a pH range of 6 - 7.0 and* A. fumigatus* LMLPS 13-1 produced *β*-glucosidase activity of 23.5 U/ml over a pH range of 6.5 - 7.0 (Figure 4). The lowest *β*-glucosidase activity produced was 20.6 U/ml by* T. longibrachiatum* LMLSAUL 14 - 1.

**Figure 5:**
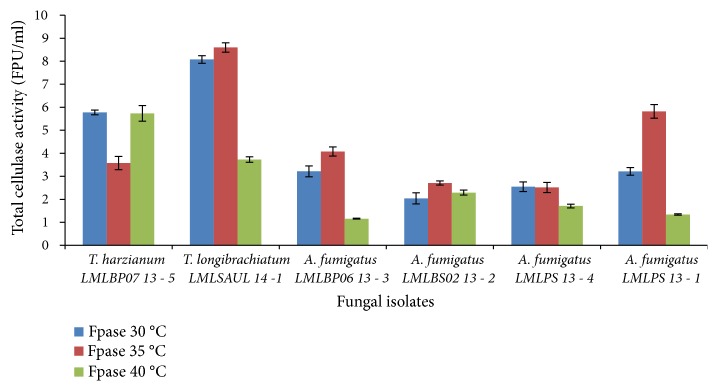
Effect of incubation temperature on the production of cellulase (total cellulase activity) by fungal strains at initial medium pH 6.5.

**Figure 6:**
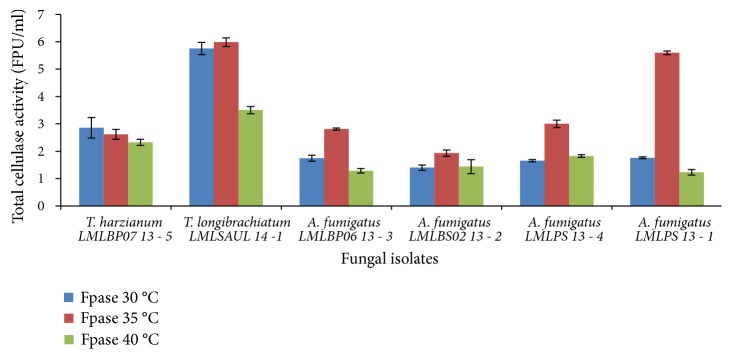
Effect of incubation temperature on the production of cellulases (total cellulase activity) by fungal strains at initial medium pH 7.0.

The production of cellulases with respect to changes in incubation temperature was also investigated at initial medium pH of 6.5 and 7.0 (Figures 5 and 6). These pH values have shown to favour production of *β*-glucosidase by species of* Aspergillus* and* Trichoderma*. At initial medium pH of 6.5 an increase in growth temperature led to a decrease in the level of total cellulase activity for* T. longibrachiatum* LMLUL 14-1 at 35°C (Figure 5). The* A. fumigatus* strains were able to produce cellulase at both 30 and 35°C, with the exception of* T*.* harzianum* LMLUL 13-5. Cellulase activity in* A. fumigatus* LMLUL 13-1 increased by 1.8-fold at 35°C (Figure 5). Increasing the incubation temperature to 40°C drastically reduced the cellulase production for* T*.* longibrachiatum* LMLUL 14-1,* A*.* fumigatus* LMLUL 13-1, and* A*.* fumigatus* LMLUL 13-3 (Figure 5). Generally, most fungal species showed cellulase activity levels decreasing as the temperature increased to 40°C.

At initial medium pH of 7.0 and a temperature of 30°C favoured high production of cellulase by* T*.* harzianum* LMLBP07 14-5,* T*.* longibrachiatum* LMLUL 14-1, and* A*.* fumigatus* LMLBS02 13-2 (Figure 6).

**Figure 7:**
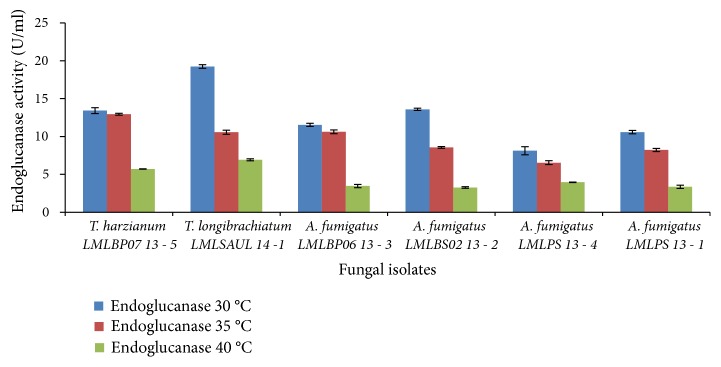
Effect of incubation temperature on the production of endoglucanase by fungal strains at initial medium pH 6.5.

**Figure 8:**
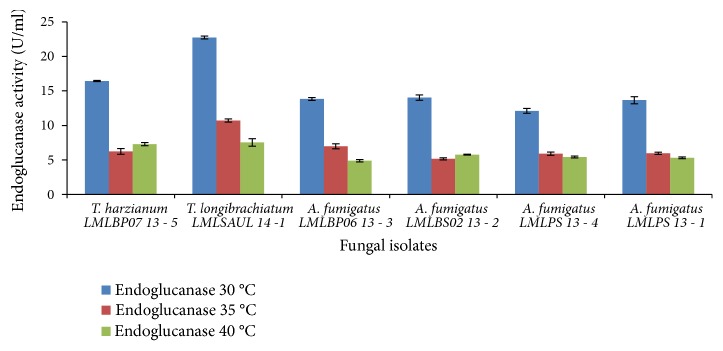
Effect of incubation temperature on the production of endoglucanase by fungal strains at initial medium pH 7.0.

The cellulase production by* A*.* fumigatus* LMLPS 13-1,* A*.* fumigatus* LMLBP06 13-3, and A.* fumigatus* LMLPS 13-4 improved at 35°C, irrespective of initial pH tested (Figures 5 and 6). At production temperature 40°C, the production of cellulases was reduced for all strains tested. All the* Trichoderma* and* Aspergillus* strains produced more endoglucanases at 30°C with both initial medium pH of 6.5 and 7.0 (Figures 7 and 8), but initial medium pH of 6.5 favoured more enzyme production. An increase in temperature to 35°C and 40°C resulted in a 2-fold decrease of the endoglucanase activity in all fungal species (Figure 7).

In an experiment with initial medium pH 7.0 a greater improvement in the production of endoglucanases was seen at 35°C (Figure 8).* T*.* harzianum* LMLBP07 13-5 produced 2-fold higher endoglucanase activity at initial medium pH of 7.0, while other fungal species attained slight increases (Figure 8).

At both initial medium pH of 6.5 and 7.0 higher temperatures caused a reduction in endoglucanase activity and severe reduction occurred at 40°C as illustrated in Figures 7 and 8.

**Figure 9:**
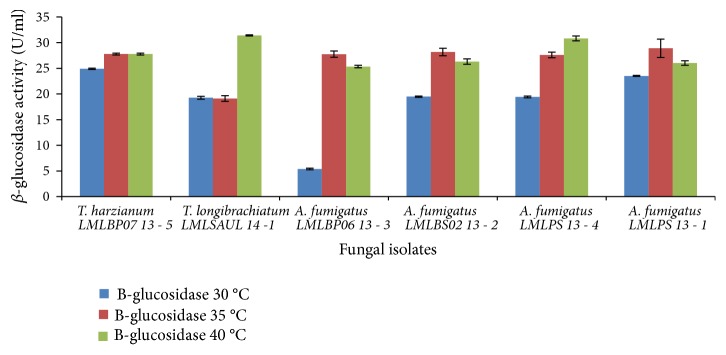
Effect of incubation temperature on the production of *β*-glucosidase by fungal strains at initial medium pH 6.5.

The production of *β*-glucosidase at an initial pH of 6.5 and 7.0 was dependent on the fungal species and production temperature. In a medium with an initial pH of 6.5, only* A*.* fumigatus* LMLPS 13-1 and* T*.* harzianum* LMLBP07 13-5 produced high levels of *β*-glucosidase activity 24.0 U/ml and 25.0 U/ml, respectively at 30°C. An increase in temperature from 30 to 40°C led to improved production of *β*-glucosidase by some fungal species. For instance,* A. fumigatus* LMLBP06 13-3 showed 5-fold increase, i.e., from 5.4 to 25.4 U/ml of *β*-glucosidase activity at 40°C and 5.2-fold increase of *β*-glucosidase at 35°C (Figure 9).

**Figure 10:**
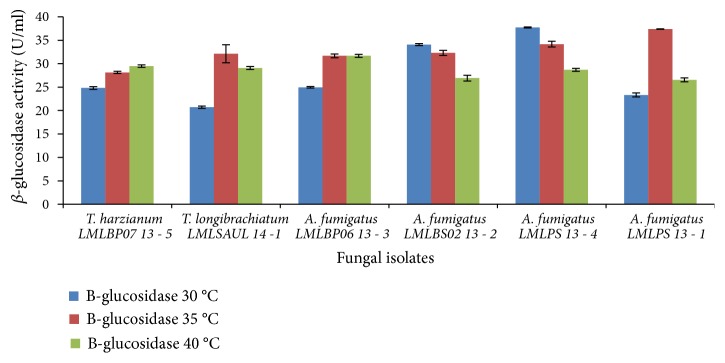
Effect of incubation temperature on the production of *β*-glucosidase by fungal strains at initial pH 7.0.

The production of *β*-glucosidase by* T. harzianum* LMLBP07 13-5 was optimal between 35 and 40°C, whilst* T*.* longibrachiatum* LMLSAUL 14-1 produced *β*-glucosidase optimally at 40°C.* A. fumigatus* LMLPS 13-4 attained maximum *β*-glucosidase activity at 30°C (Figure 9). Generally, at 40°C the production of *β*-glucosidase by all fungal species was much higher than at 30°C with the exception of* A. fumigatus* LMLPS 13-4. In a medium with initial pH 7.0, an increase in temperature led to improvement in the production of *β*-glucosidase (Figure 10). A maximum level of *β*-glucosidase level was attained at 35°C with* T. longibrachiatum* LMLSAUL 14-1 and* T. harzianum* LMLBP07 13-5 showing 1.55-fold increase and 1.13-fold increase, respectively.* A. fumigatus* LMLPS 13-4 and* A. fumigatus* LMLPS 13-1 also showed an increase of *β*-glucosidase activity at 35°C (1.76-fold and 1.6-fold, respectively).* A. fumigatus* LMLBP06 13-3 showed a proportional increase of 1.27-fold with maximum *β*-glucosidase produced at 40°C. Conversely, 40°C led to a 1.26-fold decrease of *β*-glucosidase produced by* A. fumigatus* LMLBS02 13 - 2 (Figure 10).

Generally, *β*-glucosidase activity was the highest at 35°C and pH 7.0 for all strains tested.

## 4. Discussion

### 4.1. Fungal Screening

Fungi and bacteria are associated with soil and decaying plant materials. In nature, fungi colonise the plant debris and in symbiotic relationship with other microorganisms, they secrete an assortment of proteins and a complex of hydrolytic enzymes to hydrolyse plant polysaccharides for their survival. However, not all the microorganisms are able to secrete significant amount of hydrolytic enzymes for biotechnological applications. Hence, there is a need to screen and select hypersecreting hydrolytic enzymes. In this study, three different methods for screening for cellulolytic activity, (1) microbial growth on cellulose agar, (2) clearing of cellulose in agar, and (3) reducing sugar production (or glucose), were performed. Growth on cellulose containing agar was useful for isolation of cellulolytic fungal strains. Ten fungal strains were selected based on fast and abundant growth on Avicel agar plates. Several authors have also reported the use of microcrystalline cellulose to screen for cellulase producing microorganisms [21–23]. CMC-CR (0.01%) agar plate has been used to detect endoglucanase activity as indicated by the presence of a pale yellow zone around the colony. According to Yoon* et al*. [13], the plate screening methods with dye coupled substrates provides a relatively straight forward and easy tool for specific detection of fungi that produce endoglucanase. Larger halos are the results of higher enzyme activity [2]. However, at high CR dye concentrations, fungal growth is suppressed.

### 4.2. Fungal Identification

The application of rDNA genes for identification of fungal species is based on the detection of conserved sequences in 5.8S rDNA and 28S rDNA that enables the amplification of the ITS2 region between them [24]. The identification of the fungal species based on the amplification of the ribosomal ITS region of the ribosomal DNA revealed that the organisms are* A. fumigatus* strains,* T. harzianum,* and* T. longibrachiatum*. The ITS regions are regarded as primary DNA barcode for the fungal kingdom and exhibit high reliability [25].

### 4.3. Cellulases Production and Optimisation

Subsequent to screening is process optimisation with various factors such as nutrient requirements, temperature, pH, and agitation speed. been investigated (Gautam* et al*., 2010; [26]). In this study, the effect of initial pH and temperatures on the production levels of cellulase were investigated. Fungal strains belonging to* Trichoderma* and* Aspergillus* exhibited the potential to produce cellulase over a pH 4.5-7.0 and temperature (30-40) range, although the cellulase production levels varied from one fungal strain to another under the conditions used. It has been reported that expression of fungal genes is regulated by extracellular pH and many fungi exhibit growth and enzyme secretion over a wide pH range [27]. Temperature also influences microbial growth and enzyme production [20, 28] and an optimal environment conducive to the production of cellulase will also be influenced by media nutritional composition [26].

The initial pH of 4.5-5.5 drastically reduced the levels of cellulases and endoglucanases produced by all strains. This implied that acidity of the medium possibly affected fungal growth and metabolism. Acidic medium pH effect on the production of cellulases by* Trichoderma* and* Aspergillus* was also reported by Gautam* et al*. [29] and Delabona* et al*. [23]. Contrary to these results, other authors reported such acidic condition to be favourable for production of cellulases [22, 30–34]. Our results showed that a less acidic medium with a pH 5.5 and above favoured the production of cellulases by all* Trichoderma* and* Aspergillus* strains with optimum production occurring at a pH of 6.5. Beyond pH 6.5 a drastic decrease in cellulases levels was observed. Gilna & Khaleel [35] and Gautam* et al*. ([29]; 2010) also reported maximum production of cellulases at pH 6.5. Aboul-Fotouh* et al*. [36] reported maximum production of cellulases by* Aspergillus niger* at initial medium pH of 6 when cultured on 10% rice straw. Other findings by Ncube* et al*. [37] showed no significant differences in the production of endoglucanases by* A*.* niger* FGSCA 733 over a pH range of 3-7. These findings illustrate that an optimum pH for maximum production of cellulase is dependent on fungal species and to some extent on the particular strain evaluated.

There was effect of temperature on the production of cellulases. The production of cellulases decreased with increasing temperature. At 40°C a significant reduction in endoglucanases activity was observed in all strains tested (Figures 8 and 9). On the contrary, Ncube* et al*. [37] reported maximum endoglucanase activity at 40°C by* A. niger* FGSCA 733. Other studies reported optimal cellulase and endoglucanase production at 45°C [29] and 60°C [22, 38]. A temperature of 40°C did not significantly affect *β*-glucosidase production (Figures 9 and 10). Leghlili* et al*. (2013) reported the production of cellulases, endoglucanases, and *β*-glucosidase* T. longibrachiatum* (GHL) at both 30°C and 35°C, with higher production levels obtained at 35°C. Other authors reported maximum production levels of *β*-glucosidase by* T*.* longibrachiatum* at 35°C [39] and* A*.* niger* MS82 at 25°C. [30].* Aspergillus niger* MS82 also produced sufficient endoglucanases at 30°C and 35°C in acidic initial medium pH 4.0 [30].

These discrepancies with regard to the optimum initial pH and production temperature on the levels of cellulases as measured by filter paper activity (FPU/ml) can be attributed to genetic make-up of the fungal strains as a result of adaptations to different habitats. Furthermore, our reported high levels of cellulases, endoglucanases, and low level of *β*-glucosidase by the* Trichoderma* isolates and* vice versa* high levels of *β*-glucosidase by* A. fumigatus* are in agreement with the reported levels of these fungal species by Stewart and Parry [38].

## 5. Conclusion 

In conclusion, all the* Trichoderma* and* Aspergillus* strains produced substantial levels of all the three enzymes (i.e., exoglucanase, endoglucanase, and *β*-glucosidase) required for complete hydrolysis of cellulosic material.* Trichoderma* strains produced higher cellulases and endoglucanases levels while* A. fumigatus* strains produced higher *β*-glucosidase levels. The production of all the cellulases components seemed to be strongly influenced by the interactive effect of initial pH and incubation temperature on the microorganisms. Hence, the observed maximum production of cellulases by the fungi depended on the chosen initial pH of the medium and incubation temperature. The cellulases produced by these fungal strains will be assessed for efficiency in hydrolysing agricultural lignocellulose wastes, including banana pseudostem for the production of bioethanol.

## Figures and Tables

**Table 1:** Fungal isolation and screening on Avicel and CMC-Congo Red agar.

Sampling site	Isolate code no.	Isolate taxa
Banana plantation site, Allesbeeste farm, Tzaneen	^a^LML**BP**07 13-5	*Trichoderma harzianum*
	^a^LML**BP**06 13-3	*Aspergillus fumigatus*
Decomposing banana pseudostem, Allesbeeste farm, Tzaneen	^b^LML**PS** 13-1	*Aspergillus fumigatus*
	^b^LML**PS** 13-4	*Aspergillus fumigatus*
Banana dumpsite, Allesbeeste farm, Tzaneen	^c^LML**BS**02 13-2	*Aspergillus fumigatus*
Decomposing *Strelizia alba*, University of Limpopo	^d^LML**SAUL** 14-1	*Trichoderma longibrachiatum*

^a^BP refers to banana plantation site; ^b^PS refers to Pseudostem; ^c^BS refers to banana dumpsite outside plantation; ^d^SAUL refers to *Strelitzia alba* at University of Limpopo

## Data Availability

The experimental data used to support the findings of this study are available from the corresponding author upon request.
